# Quantitative Comparison of Protein Adsorption and Conformational Changes on Dielectric-Coated Nanoplasmonic Sensing Arrays

**DOI:** 10.3390/s18041283

**Published:** 2018-04-22

**Authors:** Abdul Rahim Ferhan, Joshua A. Jackman, Tun Naw Sut, Nam-Joon Cho

**Affiliations:** 1 School of Materials Science and Engineering and Centre for Biomimetic Sensor Science, Nanyang Technological University, 50 Nanyang Drive 637553, Singapore; ferhan@ntu.edu.sg (A.R.F.); jjackman@ntu.edu.sg (J.A.J.); suttun001@e.ntu.edu.sg (T.N.S.); 2 School of Chemical and Biomedical Engineering, Nanyang Technological University, 62 Nanyang Drive 637459, Singapore

**Keywords:** nanoplasmonics, localized surface plasmon resonance, label-free biosensor, near field decay, protein adsorption, human serum albumin

## Abstract

Nanoplasmonic sensors are a popular, surface-sensitive measurement tool to investigate biomacromolecular interactions at solid-liquid interfaces, opening the door to a wide range of applications. In addition to high surface sensitivity, nanoplasmonic sensors have versatile surface chemistry options as plasmonic metal nanoparticles can be coated with thin dielectric layers. Within this scope, nanoplasmonic sensors have demonstrated promise for tracking protein adsorption and substrate-induced conformational changes on oxide film-coated arrays, although existing studies have been limited to single substrates. Herein, we investigated human serum albumin (HSA) adsorption onto silica- and titania-coated arrays of plasmonic gold nanodisks by localized surface plasmon resonance (LSPR) measurements and established an analytical framework to compare responses across multiple substrates with different sensitivities. While similar responses were recorded on the two substrates for HSA adsorption under physiologically-relevant ionic strength conditions, distinct substrate-specific behavior was observed at lower ionic strength conditions. With decreasing ionic strength, larger measurement responses occurred for HSA adsorption onto silica surfaces, whereas HSA adsorption onto titania surfaces occurred independently of ionic strength condition. Complementary quartz crystal microbalance-dissipation (QCM-D) measurements were also performed, and the trend in adsorption behavior was similar. Of note, the magnitudes of the ionic strength-dependent LSPR and QCM-D measurement responses varied, and are discussed with respect to the measurement principle and surface sensitivity of each technique. Taken together, our findings demonstrate how the high surface sensitivity of nanoplasmonic sensors can be applied to quantitatively characterize protein adsorption across multiple surfaces, and outline broadly-applicable measurement strategies for biointerfacial science applications.

## 1. Introduction

Nanoplasmonic sensing is a popular surface-sensitive measurement technique for biomolecular detection, and has several compelling features including simple instrumental requirements, label-free format, and high detection sensitivity [1,2,3]. One of the most popular versions is based on the localized surface plasmon resonance (LSPR) of metal nanoparticles. LSPR refers to the collective oscillation of free electrons in the conduction band of noble metal nanoparticles that occurs upon interaction with light, leading to light extinction that is characterized by a wavelength position with maximum extinction, λ_max_ [1,4]. The evanescent electromagnetic field surrounding LSPR-active nanoparticles is highly amplified in the near vicinity of the nanoparticle surface (~5–20 nm decay length [5,6]), and the specific value of λ_max_ is sensitive to the local dielectric environment within the probing volume. The presence of a biomolecule near the sensor surface will typically cause an increase in the local refractive index that is registered as a positive Δλ_max_ shift. As the decay length of the LSPR-enhanced field is on the length scale of various classes of biomolecules, it is possible to not only detect biomolecular binding events, but also characterize structural transformations that influence the spatial proximity of adsorbed molecules to the sensor surface [7,8,9]. In turn, these capabilities have proven useful for gaining novel insights into well-studied biomacromolecular interactions involving lipid vesicles [10,11,12,13,14] and supported lipid bilayers [8,15,16,17,18]. 

Within this scope, proteins have become a popular subject of inquiry as protein adsorption onto a solid support is often accompanied by substrate-induced denaturation and resulting conformational changes. Indeed, while most nanoplasmonic sensing works involving proteins have focused on end-point detection [19,20], there is a growing number of studies that have exploited the high surface sensitivity of LSPR measurement strategies to investigate protein-protein interactions [21,22,23,24,25], protein interactions with small molecules [26,27,28,29], sugars [30,31,32], and drugs [33,34], and protein conformational changes triggered by an environmental stimuli [9]. In recent years, the utility of LSPR sensors to study protein adsorption has become greatly expanded through the introduction of indirect nanoplasmonic sensing (INPS), which involves coating plasmonic metal nanoparticles with a dielectric material of interest [35]. In addition to tuning measurement sensitivity and increasing platform stability [36], perhaps the greatest advantage of the dielectric coating is that it facilitates nanoplasmonic sensing across a wide range of materials and surface chemistries.

In one prominent example, Langhammer and co-workers employed the INPS strategy to characterize protein adsorption onto silica-coated gold nanodisks within the context of studying protein corona formation [37,38]. In addition to coating the nanoplasmonic transducer with a conformal dielectric layer, other materials can also be deposited on the sensor surface to study protein adsorption. For example, Zen et al. investigated the adsorption of bovine serum albumin (BSA) and fibrinogen onto amorphous carbon and hydrogenated amorphous carbon using nanoplasmonic sensors comprised of gold nanodisks coated with the two types of carbon materials [39]. While the extent of protein adsorption has been mainly characterized by directly relating the magnitude of the final LSPR peak shift to the amount of adsorbed protein, the LSPR measurement response per individual protein molecule can vary because the protein’s molecular mass is, on average, in a region of different field intensity [40,41,42]. Recently, we investigated the effect of temperature on BSA adsorption and denaturation onto silica-coated nanoplasmonic sensor arrays, and showed how denaturation of individual, adsorbed protein molecules affects the LSPR measurement response [43]. To date, existing LSPR studies for protein adsorption have been performed on individual substrates, and comparative measurements of protein adsorption and denaturation across multiple substrates have yet to be explored. From a measurement perspective, the challenge lies in taking into account the different surface sensitivities of the substrates, along with the different conformations of adsorbed protein molecules that occur when protein molecules interact with a particular surface of defined material composition.

Herein, we investigated human serum albumin (HSA) adsorption onto silica- and titania-coated arrays of plasmonic gold nanodisks. Understanding protein adsorption behavior on silica and titania surfaces is important considering their prevalence in biomedical applications [44,45]. HSA was selected as the model protein because it is widely studied and known to denature (via conformational changes) in the adsorbed state [46,47,48,49,50]. HSA is also a structural cognate of BSA [51,52], and this similarity provided the basis for comparing LSPR measurement data with past findings as further verification. While we previously focused on measuring the relative denaturation of individual, BSA molecules on silica-coated surfaces at low surface coverage [43], we expand the scope of our present investigation to comprehensively investigate HSA adsorption and denaturation across different surface coverage regimes and compare results obtained on dielectric-coated substrates with different surface sensitivities. For the first time, protein adsorption behavior on different surfaces are compared on the same nanoplasmonic sensing platform. In terms of their physicochemical properties, the silica and titania coatings used in this work share some similarities in that they are both hydrophilic and negatively-charged. This allowed us to highlight the effect of subtle differences in surface properties on protein adsorption behavior. Furthermore, by varying the ionic strength condition, it was possible to tune the protein-substrate interaction, especially via charge shielding of electrostatic forces [53,54,55,56,57]. In turn, this enabled us to establish an analytical framework to quantitatively compare responses across multiple substrates with different sensitivities and demonstrate the capability of the nanoplasmonic sensing technique to identify unique patterns of protein adsorption and denaturation on different surfaces, paving the way for its utilization across various application settings.

## 2. Materials and Methods 

### 2.1. HSA Preparation

Lyophilized human serum albumin (HSA) (A3782) was obtained from Sigma-Aldrich (Singapore) and was stored at 4 °C prior to use. Buffer solutions were prepared using 10 mM tris(hydroxymethyl)aminomethane (Tris) and varying concentrations of sodium chloride. All buffer solutions were adjusted to pH 7.5 with 1 M hydrochloric acid, and filtered through a membrane filter with 0.22 μm diameter pores before use. All protein samples were freshly prepared on the day of experiments by dissolving a weighed mass of lyophilized protein in the appropriate buffer solution. The molar concentrations of protein samples were determined by measuring the sample absorbance at 280 nm wavelength and taking into account the molar extinction coefficient of 36,500 M^−1^ cm^−1^ based on previous works [58,59]. The protein concentration was adjusted by dilution to 50 µM final concentration for experiments. 

### 2.2. LSPR Measurements

Ensemble-averaged LSPR measurements were performed on an Insporion XNano instrument (Insplorion AB, Gothenburg, Sweden) that was operated in transmission mode. The highly uniform sensor chips composed of silica- and titania-coated gold nanodisk arrays on glass surfaces were purchased from Insplorion AB. The sensor chips were assembled within the measurement cell with an effective circular sensing area ~2 mm in diameter and a flow depth of ~44 µm (i.e., effective sensing volume ~0.14 µL), as previously described [13]. The arrays are comprised of well-separated and randomly distributed gold nanodisks (average height and diameter of 20 and 120 nm, respectively, with a surface coverage of ~8%), which were fabricated by hole-mask lithography [60] and sputter-coated with a thin dielectric layer (thickness ~10 nm). Prior to each experiment, the sensor chips were thoroughly rinsed with 1 wt % sodium dodecyl sulfate (SDS) in water, water, and ethanol and dried with a stream of nitrogen gas. The sensor chips were then treated with oxygen plasma before being loaded into the measurement cell. For the silica-coated sensor chips, the base material is silicon nitride and the oxygen plasma treatment results in the formation of a silica layer on the sensor surface and hence the coatings are referred to as silica coatings [61]. A peristaltic pump was used to introduce liquid sample into the measurement cell at a constant flow rate of 100 µL/min. All LSPR data collection and analysis was performed using the Insplorer software package (Insplorion AB) with a time resolution of 1 Hz. The spectral resolution of the plasmon resonance, as well as its centroid position (denoted as λ_max_), was determined by high-order polynomial fitting [62].

### 2.3. QCM-D Measurements

QCM-D measurements were performed on a Q-Sense E4 instrument (Biolin Scientific AB, Stockholm, Sweden) using AT-cut crystals with sputter-coated, 50 nm-thick silica (QSX 303, Biolin Scientific AB) or titania (QSX 310, Biolin Scientific AB) layers. The crystals had a mass sensitivity constant of 17.7 ng/cm^2^Hz (Ref. [63]). Before measurement, the crystals were rinsed with 1 wt % SDS in water, water, and ethanol, dried with a stream of nitrogen gas, and treated with oxygen plasma for 1 min. Samples were introduced at a constant flow rate of 100 µL/min, as regulated by a peristaltic pump, and the temperature of the measurement cell was maintained at 25.0 ± 0.5 °C. Data was collected at the odd overtones (3rd, 5th, 7th, and 9th), and the normalized data at the 5th overtone are reported. 

## 3. Results and Discussion

### 3.1. Sensor Characterization

**Figure 1:**
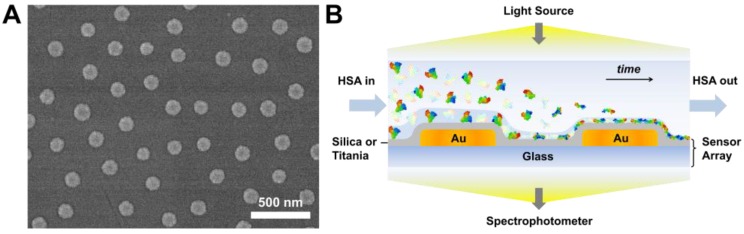
(**A**) Representative top-down SEM image of the gold nanodisk sensor array; and (**B**) a schematic illustration of HSA adsorption onto silica- and titania-coated gold nanodisk arrays.

We employed the INPS strategy to monitor HSA adsorption and denaturation on dielectric-coated sensor arrays, which were comprised of randomly-distributed gold nanodisks on glass surfaces fabricated via hole-mask colloidal lithography and the entire surface was then conformally coated with a 10-nm thick silica or titania overlayer (Figure 1A). Briefly, when HSA solution is introduced into the microfluidic chamber, HSA molecules adsorb onto the sensor surface and accumulate to gradually form a dense protein adlayer, as shown in Figure 1B. The entire process occurs under the illumination of light, which passes through the sensor to reach the spectrophotometer. The adsorption of HSA molecules onto the sensor surface leads to a Δλ_max_ shift, and our measurement approach is sensitive to the dry mass of adsorbed HSA molecules.

**Figure 2:**
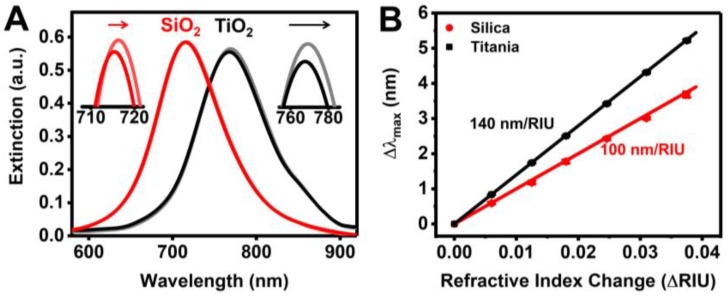
(**A**) LSPR spectra before and after HSA adsorption onto the two sensor surfaces; (**B**) Bulk refractive index sensitivity measurements from water-glycerol titration measurements (0–30 wt % glycerol). Data are reported as mean and standard deviation for *n* = 3 measurements.

The optical extinction spectra corresponding to before and after HSA adsorption onto silica- and titania-coated gold nanodisks are shown in Figure 2A. The presence of different dielectric coatings leads to a significant variation in the initial λ_max_ position (i.e., ~715 nm and ~767 nm for silica- and titania-coated sensors, respectively), which arises from perturbations in the electromagnetic field distribution around the gold nanodisks due to the presence of the dielectric coatings [64,65,66]. Consequently, the distinct plasmonic properties result in the sensor arrays exhibiting different bulk refractive index sensitivities, which typically increase as the LSPR peak position moves to higher wavelengths [67,68]. We determined the bulk refractive index sensitivities to be ~100 and ~140 nm/RIU for bare silica- and titania-coated sensors, respectively (Figure 2B). While electromagnetic field enhancement around the edges of bare plasmonic nanostructures is often amplified by up to several orders of magnitude more than flat or smooth regions [69,70,71], past simulation results suggest a relatively uniform electromagnetic field distribution around the contacting surface of dielectric-coated gold nanodisks [11]. 

After HSA adsorption under physiologically-relevant ionic strength conditions (i.e., 150 mM NaCl) for 20 min, a positive Δλ_max_ shift was observed on both surfaces. While the magnitude of the Δλ_max_ shift (~1 nm) is relatively small, it is significantly larger than the sensing resolution by several orders of magnitude. This was verified by directly characterizing the sensing resolution, which was defined as the smallest change in the bulk refractive index that produced a detectable change in the output [72]. The practical resolutions were determined to be 9.20 × 10^−5^ RIU and 1.12 × 10^−4^ RIU, which translates to Δλ_max_ shifts of around 0.009 nm and 0.016 nm, for silica- and titania-coated sensors, respectively (details in Supplementary Materials). Taken together, these findings identify that the fabricated titania-coated arrays have ~1.4-times greater measurement sensitivity than the silica-coated arrays, and establish that HSA protein molecules adsorb onto both sensor surfaces producing detectable measurement responses.

### 3.2. Real-Time Monitoring of HSA Adsorption Kinetics

**Figure 3:**
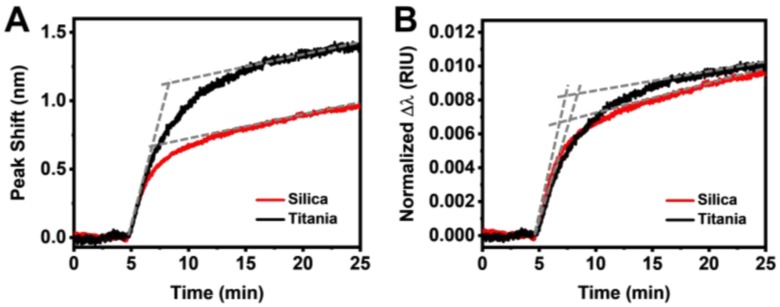
(**A**) LSPR peak shift responses as a function of time for HSA adsorption onto silica- and titania-coated sensors before normalization; and (**B**) the normalized LSPR peak shift responses as a function of time for HSA adsorption from panel (**A**).

To resolve protein adsorption kinetics, we tracked the Δλ_max_ shift as a function of time. As shown in Figure 3A, the time-resolved peak shifts indicated that HSA adsorption onto silica and titania surfaces shows similar adsorption profiles under physiologically relevant ionic strength conditions (i.e., 150 mM NaCl). In particular, there were similar adsorption rates in the initial and equilibration stages, while one distinct difference was a significantly higher final peak shift for the titania case. The final peak shifts were around 0.9 nm and 1.4 nm for HSA adsorption onto silica and titania, respectively, and the range of these values agrees well with literature values for BSA adsorption [37,38,43], validating our measurement approach. The LSPR signals can be normalized by the bulk refractive index sensitivities to directly compare the measurement responses across different substrates [11]. After normalizing the measurement responses by the bulk refractive index sensitivities, it is noticeable that the extent of HSA adsorption was nearly identical on the two substrates (Figure 3B). Specifically, the rates in the initial and equilibration stages were slightly higher for HSA adsorption onto silica than titania surfaces, with the trend switching in the intermediate stage.

**Figure 4:**
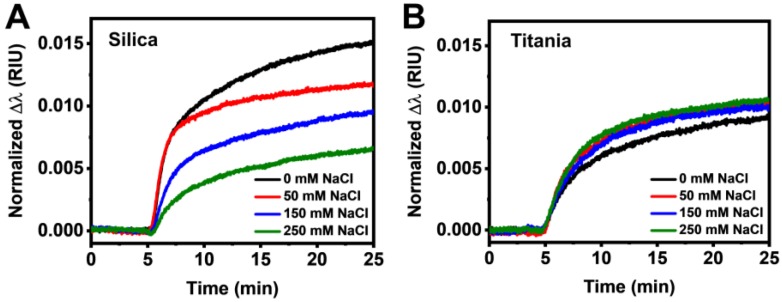
Normalized LSPR peak shift responses as a function of time for HSA adsorption onto (**A**) silica and (**B**) titania surfaces in 0, 50, 150, and 250 mM NaCl conditions.

It is known that the presence of salt can affect protein-protein and protein-substrate interactions through electrostatic shielding effects [56,73]. In addition, protein conformation can also be stabilized by the presence of salt [74,75,76,77]. Since a silica surface is more negatively charged than a titania surface under the tested pH conditions [52], we proceeded to investigate HSA adsorption onto both surfaces under different ionic strength conditions ranging from 0 mM to 250 mM NaCl to evaluate surface charge effects. Interestingly, significant differences in adsorption behavior became apparent at different ionic strengths. With increasing ionic strength, there was a decrease in the normalized peak shifts for the case of HSA adsorption onto silica, indicating lower accumulation of protein molecules within the immediate vicinity of the gold nanodisks (Figure 4A). In marked contrast to the silica case, minimal variations in the measurement response were observed for the titania case (Figure 4B).

**Figure 5:**
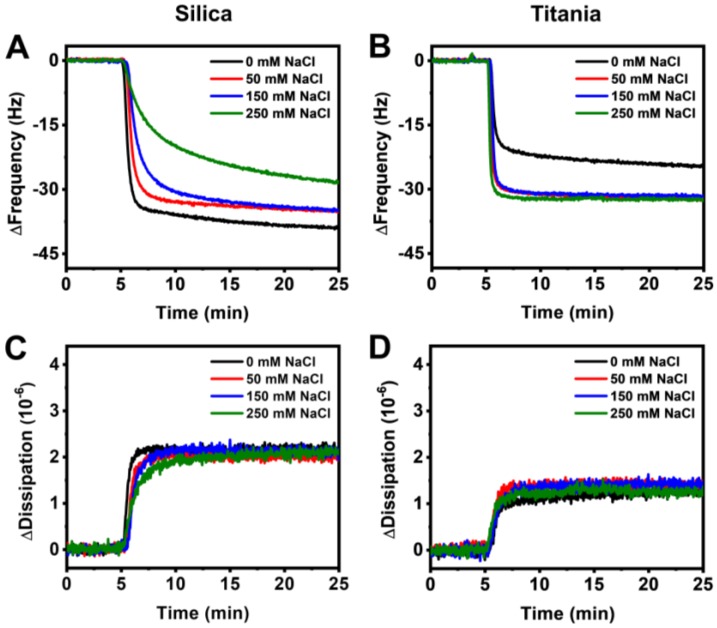
QCM-D frequency shifts for HSA adsorption onto (**A**) silica and (**B**) titania at 0, 50, 150, and 250 mM NaCl. Accompanying dissipation shifts for adsorption onto (**C**) silica and (**D**) titania.

In order to verify the observed measurement trends, we performed quartz crystal microbalance-dissipation (QCM-D) experiments using silica- and titania-coated quartz crystals. The QCM-D technique tracks changes in the resonance frequency and energy dissipation of an oscillating quartz crystal that reflect the acoustic mass and viscoelastic properties of an adsorbate, respectively. With increasing ionic strength, there was an overall decrease in the QCM-D frequency shift for the case of HSA adsorption onto silica, indicating lower accumulation of protein molecules on the surface (Figure 5A). Compared to the LSPR peak shifts, the QCM-D frequency and dissipation shifts for HSA adsorption onto silica were less varied across the tested range of ionic strength conditions. In contrast, frequency shifts closely overlapped for the case of HSA adsorption onto titania, except for the case of 0 mM NaCl, which indicated the lowest amount of adsorbed HSA (Figure 5B). The accompanying energy dissipation shifts showed similar values during the stabilization stage for HSA adsorption onto silica, suggesting that protein molecules are closely packed to form a compact film and the specific configuration likely depends on the particular ionic strength condition (Figure 5C). Likewise, energy dissipation shifts for HSA adsorption onto titania at different ionic strengths were also similar, although the values were much lower compared to the case of silica, indicating that HSA molecules are more deformed and tightly bound on the titania surface (Figure 5D). Overall, the trends observed via QCM-D measurements were in excellent agreement with those observed via LSPR measurements. 

### 3.3. Data Analysis

A detailed analysis of the LSPR response requires some prior knowledge about the biomacromolecular interaction under consideration. In general, a protein molecule initially adsorbs onto a surface in its native conformation and then, depending on the protein-surface interaction, either remains in its native conformation or unfolds and spreads on the surface [42,78,79,80]. Upon spreading, the surface area covered by a protein molecule, as well as the binding strength between the surface and protein molecule increases [81,82]. Depending on the extent of protein deformation, protein molecules can either remain adsorbed or desorb from the surface. Over time, the protein adsorption process will become less reversible and the extent of protein spreading will increase after multiple adsorption-desorption cycles [82,83,84,85,86].

When interpreting LSPR measurement responses, it is often assumed that protein density remains constant [87,88] leading to a direct correlation between LSPR peak shift and optical mass. However, as mentioned above, both protein density and thickness of the protein film change. As described in our previous works [11,13,14], the peak shift, ${\Delta \lambda }_{\max}$, arising from a refractive index change in the LSPR-probed sensing volume around the nanodisk is denoted by:

(1) $${\Delta \lambda }_{\max}=S_{B}\int_{z=0}^{\infty }\frac{5R_{*}^{5}}{{\left(R_{*}+Z\right)}^{6}}\Delta n\left(z\right)dz$$

where $S_{B}$ is the bulk refractive index sensitivity of the sensing arrays (i.e., 100 nm/RIU and 140 nm/RIU for silica- and titania-coated surfaces, respectively), z is the coordinate perpendicular to the substrate surface (z=0 corresponds to the protein-substrate), $R_{*}$ is the length scale characterizing the distance between the center of the nanodisk and the protein-substrate contact (i.e., 74 nm as previously determined by simulation results [11]), and Δn(z)dz is the spatial distribution of the refractive index change along the z coordinate. Considering the formation of a protein layer directly on the sensor surface, integration of Equation (1) yields:

(2) $$\bar{{\Delta \lambda }_{\max}}=\Delta n\left[1-{\left(\frac{R_{*}}{R_{*}+D}\right)}^{5}\right],$$

where $\bar{{\Delta \lambda }_{\max}}$ is the normalized peak shift and Δn is the refractive index change arising from the adsorption of protein molecules to form a protein layer with thickness D. We applied the above correlation to approximate the thickness of the HSA layer based on typical values of local refractive index changes due to HSA adsorption onto titania, which is in the range of 0.08 to 0.12 RIU after 30 mins [88]. Rearranging Equation (2), we obtain:

(3) $$D={\left[R_{*}^{5}{\left(1-\frac{\bar{{\Delta \lambda }_{\max}}}{\Delta n}\right)}^{-1}\right]}^{\frac{1}{5}}-R_{*},$$

Substituting the typical values of Δn, as well as $\bar{{\Delta \lambda }_{\max}}$ from Figure 4A, B into Equation (3), we obtain a protein film thickness of around 1.5 nm for adsorbed protein layers on both surfaces at physiological ionic strength condition, which agrees well with previous works [38,39]. Based on the above arguments, the LSPR measurement response (Δλ_max_ shift) can arise from either protein adsorption (mass accumulation) on the surface and/or from substrate-induced conformational changes [43,89]. Applying this framework to protein films requires a delicate approach because (1) the majority of albumin molecules will desorb shortly after they initially adsorb on the surface, especially when there is low surface coverage [90]. Over time, (2) HSA molecules that remain irreversibly adsorbed reduce the available surface area for subsequent adsorption events and, as a consequence, (3) there will gradually be a lower flux of newly adsorbing proteins [82,86]. 

Having established the framework for interpreting LSPR responses, let us first analyze the data for HSA adsorption on silica and titania at physiological ionic strength. By comparing the time-resolved peak shifts in Figure 3B, we can deduce that the initial rate of reversible adsorption was higher on silica, while the tendency for protein spreading is lower. Consequently, protein spreading on silica occurred more gradually over a longer time period. On the other hand, a quicker and higher degree of HSA spreading occurred on titania. The evidence supports that, due to a higher surface charge, the initial adsorption of HSA on silica is more strongly governed by long-range electrostatic interactions [91,92], compared to that on titania where the adsorption process is less driven by electrostatic forces (i.e., no dependence on ionic strength) [93,94]. Conversely, this supports that HSA spreading on titania occurs due to stronger, short-range protein-surface interactions stemming from non-electrostatic forces (e.g., van der Waals). Furthermore, the LSPR measurements also showed a lower propensity for HSA desorption from titania upon rinsing with buffer (Figure S1). Of note, Givens et al. recently showed that BSA adsorption onto oxide nanoparticles follows a similar trend, whereby adsorbed BSA is more completely denatured on titania nanoparticle surfaces, while it is less denatured on silica nanoparticle surfaces [95]. 

Furthermore, the decrease in initial rate and equilibration uptake with increasing ionic strength on silica suggested lower rates of reversible adsorption and greater spreading. Interestingly, for 0 mM and 50 mM NaCl conditions, the initial rates were closely matching before the rate decreased more considerably for 50 mM NaCl during the intermediate stage. This result is reasonable since it is known that proteins are destabilized in the absence of salt due to the lack of intramolecular charge shielding resulting in greater intramolecular repulsions [74,75,77]. The observations also suggest that HSA spreading and/or reorientation in the later stage was induced by further protein uptake, which did not occur for 50 mM NaCl since the adsorbed HSA molecules were more stable and, therefore, had lesser tendency to reorient to accommodate later arriving molecules [96]. In contrast, HSA adsorption onto titania did not change significantly as compared to adsorption under physiological ionic strength, supporting that HSA adsorption onto titania is strongly governed by short-range interaction forces. Compared to HSA desorption from silica, HSA desorption from titania was also lower at all tested ionic strengths (Figure S2). In addition, stronger van der Waals forces on titania also promote the formation of a thicker hydration layer [97], which may mediate the propensity for protein deformation on the surface, resulting in similar rates of conformational change on the surface independent of ionic strength. 

Taken together, our results reveal that, while HSA adsorption onto two hydrophilic surfaces appears to be very similar under physiologically relevant ionic strength conditions, significant variations in adsorption behavior are observed under different ionic strength conditions and can be detected by LSPR measurements. Comparison between experimentally-measured HSA adsorption behaviors on silica and titania surfaces is briefly summarized in Table 1, and provides insight into the role of different factors, as well as benefits of the INPS sensing approach for measurement discrimination.

## 4. Conclusions

In this work, we have employed a nanoplasmonic sensing technique to comparatively investigate HSA adsorption onto silica and titania surfaces. This represents the first work comparing protein adsorption behavior on different surfaces using the same nanoplasmonic sensing platform. We highlight the capability of the technique to clearly resolve differences in HSA adsorption behavior on two surfaces that share similar physicochemical properties in that they are both hydrophilic and negatively charged. By comparing time-resolved LSPR peak shifts at different ionic strength conditions, we revealed that the amount of adsorbed HSA and the degree of protein spreading on a silica surface was highly dependent on the ionic strength condition, while HSA adsorption behavior on a titania surface was largely unaffected by changes in ionic strength. Data analysis further underscored the particular significance of the high surface sensitivity of the nanoplasmonic sensing approach to distinguish the ionic strength-dependent trends. In general, our results suggest a higher degree of protein spreading on titania. Based on these findings, we concluded that the HSA adsorption onto silica is mainly governed by long-range electrostatic interactions and adsorbed HSA molecules would experience weaker short-range interactions than on titania. In the latter case, it appears that non-electrostatic forces (e.g., van der Waals) and surface hydration have more significant effects on influencing protein adsorption on titania than electrostatic forces alone. Taken together, our findings demonstrate how nanoplasmonic sensors can be applied to identify unique patterns of protein adsorption and denaturation on different sensor surfaces, and offer unique measurement capabilities for characterizing protein adsorption and conformational changes at solid-liquid interfaces. 

## Figures and Tables

**Table 1:** Summary of the differences in surface properties between silica and titania and the resulting differences in measurement responses obtained from QCM-D and LSPR measurement techniques.

	Silica	Titania
Surface charge (influenced by hydroxyl groups)	**Highly negative**	**Slightly negative**
Force governing protein-surface interaction	**Electrostatic**	**Non-electrostatic/van der Waals**
Degree of surface hydration	**Low**	**High**
Dependency of protein adsorption on ionic strength based on QCM-D responses	**Weak**	**Weak**
Dependency of protein adsorption on ionic strength based on LSPR responses	**Strong**	**Negligible**

## Supplementary Materials

The following are available online at http://www.mdpi.com/1424-8220/18/4/1283/s1, Information on the Approximation of Protein Layer Thickness, Determination of Practical Sensor Resolution, and Supporting Figures S1–S2.
