# Supplementary material for: Quantitative Comparison of Protein Adsorption and Conformational Changes on Dielectric-Coated Nanoplasmonic Sensing Arrays
Source: Sensors (Basel). 2018 Apr 22;18(4):1283. doi: 10.3390/s18041283 (PMC5948918; doi:10.3390/s18041283)
Supplement: Supplementary file 1 [file sensors-18-01283-s001.zip › sensors-287756-SI.pdf]

Supplementary Data

Quantitative Comparison of Protein Adsorption and Conformational Changes on Dielectric-Coated Nanoplasmonic Sensing Arrays

Abdul Rahim Ferhan [1], Joshua A. Jackman [1], Tun Naw Sut [1], and Nam-Joon Cho [1,2,*]

[1] School of Materials Science and Engineering and Centre for Biomimetic Sensor Science, Nanyang Technological University, 50 Nanyang Drive, 637553, Singapore; ferhan@ntu.edu.sg (A.R.F.); jjackman@ntu.edu.sg (J.A.J.); suttun001@e.ntu.edu.sg (T.N.S.)
[2] School of Chemical and Biomedical Engineering, Nanyang Technological University, 62 Nanyang Drive, 637459, Singapore
* Correspondence: njcho@ntu.edu.sg; Tel.: +65-6790-4925

Approximation of Protein Layer Thickness

The thickness of the adsorbed protein layer on the sensor arrays can be approximated from the measured peak shift based on the following correlation established in our previous works [1-3].

The peak shift, $\Delta\lambda_{max}$, arising from a refractive index change in the LSPR-probed sensing volume around the nanodisk is denoted by

$$\Delta\lambda_{\max} = S_B \int_{z=0}^{\infty} \frac{5R_*^5}{(R_*+Z)^6} \Delta n(z)\, dz, \tag{1}$$

where S_B is the bulk refractive index sensitivity of the sensing arrays (*i.e.*, 100 nm/RIU and 140 nm/RIU for silica- and titania-coated sensing arrays, respectively), z is the coordinate perpendicular to the substrate surface ($z = 0$ corresponds to the protein-substrate), R_* is the length scale characterizing the distance between the center of the nanodisk and the protein-substrate contact (*i.e.*, 74 nm as previously determined by finite-difference time-domain simulation results [1]), and $\Delta n(z)dz$ is the spatial distribution of the refractive index change along the z coordinate. Considering the formation of a protein layer directly on the sensing surface, Integration of Eq. 1 gives

$$\Delta\lambda_{\max} = S_B \Delta n \left[1 - \left(\frac{R_*}{R_*+D}\right)^5\right], \tag{2}$$

where Δn is the refractive index change arising from the adsorption of proteins to form a protein layer with thickness D.

Eq.2 can be rearranged and expressed in terms of normalized peak shift, $\overline{\Delta\lambda_{\max}}$, as follows

$$\frac{\Delta\lambda_{\max}}{S_B} = \Delta n \left[1 - \left(\frac{R_*}{R_*+D}\right)^5\right],$$

$$\overline{\Delta\lambda_{\max}} = \Delta n \left[1 - \left(\frac{R_*}{R_*+D}\right)^5\right], \tag{3}$$

Eq.3 can be further rearranged and expressed in terms of protein layer thickness, D, as follows

$$D = \left[R_*^5 \left(1 - \frac{\overline{\Delta\lambda_{\max}}}{\Delta n}\right)^{-1}\right]^{\frac{1}{5}} - R_*, \tag{4}$$

In this case, since the adsorption of HSA on the sensing surface occurred in buffer,

$$\Delta n = n_{\text{HSA}} - n_{\text{buffer}}, \tag{5}$$

where n_{HSA} refers to the refractive index of the protein layer, which was taken to be around 1.44, as reported previously [4], and n_{buffer} refers to the refractive index of the buffer, which was measured using an Abbe refractometer and determined to be 1.336. The protein layer thickness, D, can therefore be approximated by substituting these values, along with the value of R_* and the experimentally determined $\overline{\Delta\lambda_{\max}}$ values.

Determination of Practical Sensor Resolution

Following the approach established by Homola [5], the practical sensor resolution is defined as

$$r_{\text{RI}} = \frac{\sigma_{\text{SO}}}{S_B}, \tag{6}$$

where σ_{SO} is the standard deviation of noise of the sensor output and S_B is the bulk refractive index sensitivity of the sensor array (*i.e.*, 100 nm/RIU and 140 nm/RIU for silica- and titania-coated sensing arrays, respectively). The standard deviation of noise obtained from the baseline during HSA adsorption at physiological ionic strength was found to be at 0.00920 nm and 0.01567 nm, for silica- and titania-coated sensors, respectively. The practical sensor resolutions were therefore calculated to be 9.20×10^{-5} RIU and 1.12×10^{-4} RIU, for silica- and titania-coated sensors, respectively.

Supporting Figures

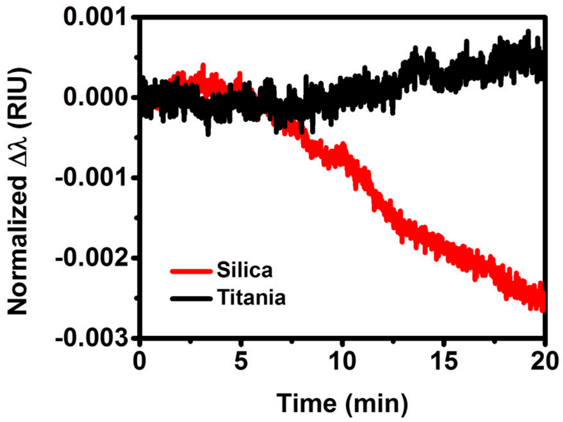

Figure S1. Normalized LSPR peak shift responses vs time as a result of HSA desorption from silica and titania at 150 mM NaCl after rinsing with buffer.

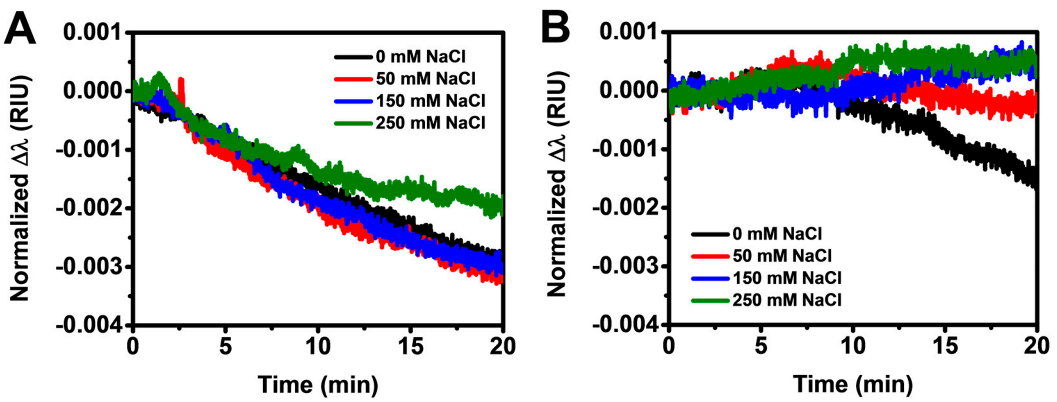

Figure S2. Normalized LSPR peak shift responses vs time as a result of HSA desorption from **(A)** silica and **(B)** titania at 0, 50, 150 and 250 mM NaCl after rinsing with buffer.

References

1. Jackman, J. A.; Spackova, B.; Linardy, E.; Kim, M. C.; Yoon, B. K.; Homola, J.; Cho, N.-J., Nanoplasmonic ruler to measure lipid vesicle deformation. *Chem. Commun.* **2016,** 52, (1), 76-79.
2. Jackman, J. A.; Zhdanov, V. P.; Cho, N.-J., Nanoplasmonic biosensing for soft matter adsorption: kinetics of lipid vesicle attachment and shape deformation. *Langmuir* **2014,** 30, (31), 9494-9503.
3. Oh, E.; Jackman, J. A.; Yorulmaz, S.; Zhdanov, V. P.; Lee, H.; Cho, N.-J., Contribution of temperature to deformation of adsorbed vesicles studied by nanoplasmonic biosensing. *Langmuir* **2015,** 31, (2), 771-781.
4. Vörös, J., The density and refractive index of adsorbing protein layers. *Biophys. J.* **2004,** 87, (1), 553-561.
5. Homola, J., Surface plasmon resonance sensors for detection of chemical and biological species. *Chem. Rev.* **2008,** 108, (2), 462-493.
